# Multi-pass microscopy

**DOI:** 10.1038/ncomms12858

**Published:** 2016-09-27

**Authors:** Thomas Juffmann, Brannon B. Klopfer, Timmo L.I. Frankort, Philipp Haslinger, Mark A. Kasevich

**Affiliations:** 1 Physics Department, Stanford University, 382 Via Pueblo Mall, Stanford, California 94305, USA; 2 Department of Physics, University of California–Berkeley, 366 Le Conte Hall MS 7300, Berkeley, California 94720, USA

## Abstract

Microscopy of biological specimens often requires low light levels to avoid damage. This yields images impaired by shot noise. An improved measurement accuracy at the Heisenberg limit can be achieved exploiting quantum correlations. If sample damage is the limiting resource, an equivalent limit can be reached by passing photons through a specimen multiple times sequentially. Here we use self-imaging cavities and employ a temporal post-selection scheme to present full-field multi-pass polarization and transmission micrographs with variance reductions of 4.4±0.8 dB (11.6±0.8 dB in a lossless setup) and 4.8±0.8 dB, respectively, compared with the single-pass shot-noise limit. If the accuracy is limited by the number of detected probe particles, our measurements show a variance reduction of 25.9±0.9 dB. The contrast enhancement capabilities in imaging and in diffraction studies are demonstrated with nanostructured samples and with embryonic kidney 293T cells. This approach to Heisenberg-limited microscopy does not rely on quantum state engineering.

## Abstract (web summary)

Low-damage and high-precision imaging can be achieved by passing the same probe photons through the specimen more than once, and this has been previously achieved in double-pass transmission microscopy. Here, the authors generalize this idea to full-field multi-pass microscopy using a self-imaging cavity.

## Graphical abstract

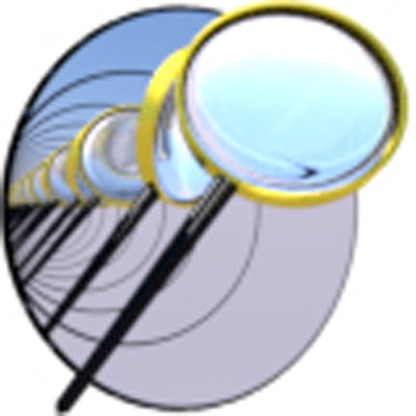

Quantum enhanced metrology allows sub-shot noise measurements by exploiting quantum correlations between probe particles^1^. This has been demonstrated in microscopy in scanning configurations applying N00N states^2^,^3^ or squeezed light^4^. Full-field shadow imaging was demonstrated using entangled photons from parametric down-conversion^5^. Experimentally, these studies relied on postselection and the reduction in variance was <3.3 dB, mainly due to the difficulties in creating the necessary correlations between the photons. On the other hand, it has also been shown that, under conditions of equivalent sample damage, a single probe particle that interacts *m* times sequentially with the sample can be used to reach the same (Heisenberg) noise limit, and that this represents an optimal parameter estimation strategy^6^,^7^,^8^. In this way a variance reduction of >10 dB was achieved in a phase-shift measurement^9^. Contrast enhancement in full-field double-pass transmission microscopy was demonstrated using a phase-conjugated mirror to pass light twice through a sample^10^.

In the following, we generalize these techniques to full-field multi-pass microscopy by placing a sample in a self-imaging cavity^11^,^12^,^13^. The setup allows us to form an image of enhanced contrast by re-imaging a pulse of light *m* times onto the sample. Although continuous wave cavity-enhanced techniques have increased measurement resolution in various fields of science, recently, for example, at LIGO^14^ or in a scanning cavity microscope^15^, counting the exact number of interactions allows for a precise parameter estimation and an enhanced sensitivity also for phase shifts larger than 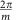. At a constant number of photon sample interactions and employing a temporal postselection scheme, we show both retardance and transmission measurements with a sensitivity beyond the single-pass shot-noise limit, which we define as the resolution obtained in our setup using only a single pass through the sample. It is limited by the shot noise on the number of detected photons. We show micrographs of nanostructured and biological samples, as well as the signal-enhancing capabilities in diffraction studies.

## Results

### The setup

**Figure 1:**
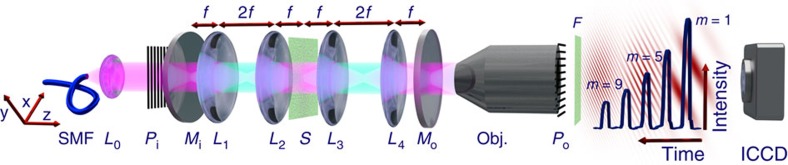
Sketch of the self-imaging cavity. A pulse of light (indicated in magenta) from a single-mode fibre (SMF) is collimated and enters the self-imaging cavity through the in-coupling mirror *M*_i_. The lenses *L*_1_ and *L*_2_ (*L*_3_ and *L*_4_, focal length *f*) form a microscope on the left (right) side of the sample *S*. Light scattered in the sample plane is indicated in turquoise. After each interaction an image is formed on either *M*_i_ or *M*_o_ and reimaged onto the sample. At *M*_o_, a fraction of the light is out-coupled and imaged using a microscope objective (Obj.) onto a gated camera (ICCD). Diffraction patterns can be imaged in the Fourier plane *F*. For polarization microscopy crossed polarizers are added (*P*_i_ and *P*_o_). The blue trace shows the integrated detected intensity as a function of delay, overlaid with simulated images corresponding to the respective intensity peaks.

Our setup is depicted in Fig. 1. A pulse of light (see Methods) is coupled into the cavity via the in-coupling mirror *M*_i_. Four lenses *L*_1–4_ form a microscope on either side of the sample. After the first light–sample interaction, an image is formed on the out-coupling mirror *M*_o_. Most of the light is reflected back onto the sample, which is now illuminated by an image of itself. An image of enhanced contrast will then be formed on *M*_i_ and will again be reflected onto the sample. This process is repeated multiple times. Every time an image is formed on *M*_o_, a fraction of the light is out-coupled and imaged onto a gated intensified CCD (charge-coupled device) camera. The gating time of the CCD camera is much shorter than the cavity roundtrip time (<500 ps and 2.7 ns, respectively), such that light can be postselected that interacted with the sample exactly *m* times.

### Polarization microscopy

**Figure 2:**
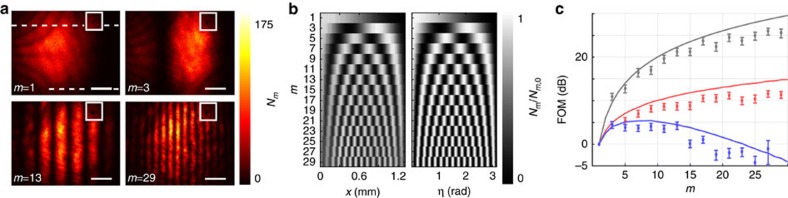
Sub shot-noise polarization microscopy. (**a**) Micrographs after *m*=1,3,13 and 29 interactions (scale bar, 250 μm) with the quartz-silica depolarizer. The inset data are taken without crossed polarizer and shows a dark spot, probably a piece of dust, reimaged onto itself *m* times. (**b**) The measured number of photons (normalized) per column (left) agrees well with the expected signal (right). (**c**) Experimental and calculated (solid lines) variance reduction in multipass microscopy for different numbers of collected photons (see text). The error bars give the s.d. of the variance (see Methods).

To demonstrate contrast enhancement and sub-shot-noise imaging, a wedged quartz-silica depolarizer is placed in the sample plane S. Every interaction with the quartz crystal leads to a position-dependent rotation of the polarization vector on the Poincaré sphere. For a properly cut and oriented quartz crystal (see Methods), the detected number of photons in a cross-polarized setup is expected to be 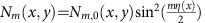, where *N*_*m*,0_(*x*, *y*) is the number of photons detected without the polarization analyser *P*_o_. *η*(*x*) is the retardance of the sample, which is proportional to the local thickness of the wedged quartz crystal. Although on a single interaction the transmitted intensity varies slowly across the field of view, more rapid signal oscillations are observed for *m*=3, 13 and 29 interactions (Fig. 2a). It is noteworthy that the ambiguity that arises from having *m* fringes instead of a single fringe can be resolved by acquiring images for different numbers of interactions.

For each image in Fig. 2a, a total number of *N*_*m*_=∑_*x*_∑_*y*_*N*_*m*_(*x*, *y*)=1.4 × 10^6^ photons were detected. Figure 2b compares the measured (left) and calculated (right) ratio ∑_*y*_*N*_*m*_(*x*, *y*)/∑_*y*_*N*_*m*,0_(*x*, *y*), where the summation is carried out in between the dashed lines in Fig. 2a. The visibility of the intensity modulation is reduced from 0.98 after 1 interaction to 0.52 after 29 interactions. As a figure of merit (FOM) for multi-pass microscopy, we define the reduction in variance of the retardance measurement 
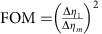
, where Δ*η*_*m*_ is the s.d. of *η*_*m*_, which can be obtained from the measured s.d. of *N*_*m*_(*x*)=∑_*y*_*N*_*m*_(*x*, *y*) via error propagation as 
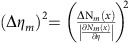
. The FOM at 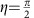 is plotted in Fig. 2c (grey data points) and shows a variance reduction by 25.9±0.9 dB after 27 interactions. This is slightly lower than the theoretically expected value FOM=*m*^2^ (grey line, see Methods) due to residual birefringence of the optical setup, reflections and misalignment. This variance reduction has been obtained for a constant number of detected photons. It is the relevant FOM if the accuracy of a measurement is limited by a finite detection rate, for example, due to the dead time of a detector. Assuming a low loss setup, it is also the relevant FOM if the number of detected photons is limited by either the collection and detection efficiency or by the number of available probe particles.

Multi-pass microscopy also offers an advantage when the number of probe particle–sample interactions has to be limited due to probe particle-induced damage. Under this constant damage condition, assuming a lossless cavity and sample, a FOM=*m* is expected (red line, see Methods). The total number of detected photons now depends on the number of roundtrips 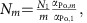 such that the total number of interactions is independent of *m*. This implies an *m*-fold damage reduction if Δ*η* is kept constant. *α*_Po,*m*_ gives the fraction of light blocked by the polarization analyser *P*_o_ and is close to 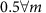.The measurements (red data points) show a maximum variance reduction of 11.6±0.8 dB, which is observed after 25 interactions. It is noteworthy that under the assumption of constant damage, the measurement error for a single multi-passed particle is equivalent to the error obtained in a Heisenberg-limited measurement with *m* entangled particles and a single pass.

Photon loss due to absorption, reflection or scattering in the sample (*α*_s_=1−*t*_s_=0.039 per interaction) or in the cavity (*α*_c_=1−*t*_c_=0.189 per interaction) reduce the efficiency of multi-pass microscopy, as only a total number of 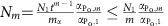 photons will be detected for operation at constant damage. Here, *t*=*t*_c_*t*_s_=1−*α* and *m*_*α*_<*m* is the mean number of interactions of a photon with the sample before it is either lost or the *m*th interaction is reached (see Methods). Taking this into account, a maximum variance reduction of 4.4±0.8 dB after three interactions is observed (blue data). This could be improved significantly in an optimized setup, as the losses in the cavity optics are mainly due to the finite reflectivity of the in- and out-coupling mirror, which, for this proof-of-principle experiment, were chosen to out-couple a considerable fraction of the beam intensity after every interaction.

### Transmission measurements and diffraction studies

**Figure 3:**
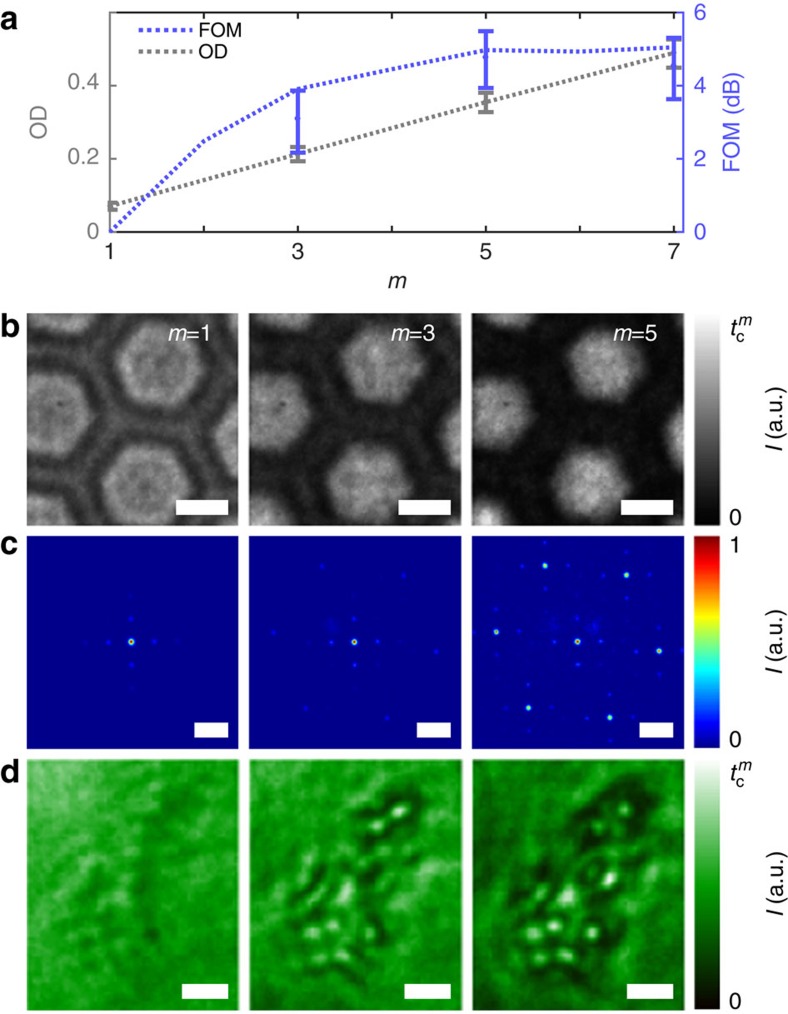
Contrast enhancement in absorption microscopy and diffraction. (**a**) Photon counting measurements of an optical density (OD) filter show a linear growth of the OD with *m* and a variance reduction at constant damage of up to 4.8±0.8 dB (see Methods). (**b**) Contrast enhancement in multi-pass micrographs of a micro-structured carbon membrane (normalized to 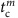) as well as in the respective diffraction patterns (**c**) (normalized to the central peak). (**d**) Multi-pass micrographs of embryonic kidney 293T cells. Scale bar, 20 μm, 10 mm^−1^ and 20 μm for **b**–**d**, respectively.

Transmission measurements can also benefit from multi-passing as long as the total losses due to the sample and the cavity are small (see Methods). Measuring the optical density of a spatially homogeneous sample shows signal amplification and a variance reduction by 4.8±0.8 dB (Fig. 3a, again at constant damage). Owing to the photon losses in the sample and in the cavity no further decrease in variance is observed when the number of interactions is increased to beyond 7.

The imaging and contrast-enhancement capabilities of the setup are further exemplified with microfabricated grating structures and with embryonic kidney 293T cells. Figure 3b shows multi-pass (*m*=1, 3, 5) micrographs of a hexagonal hole pattern in a carbon membrane. The resolution of the microscope is ∼5 μm due to the finite numerical aperture of the employed lenses (*f*=50 mm). The photon loss due to the carbon membrane is amplified by multiple passes, which leads to a significant contrast enhancement. This also becomes apparent in the images shown in Fig. 3c, which were taken in the Fourier plane of the imaging optics. Although the single pass image is dominated by the diffraction pattern caused by the four-sided copper support structure of the carbon membrane, the hexagonal symmetry of the holes in the membrane becomes clearly visible after multiple interactions. In addition, for embryonic kidney 293T cells a clear contrast enhancement is observed (Fig. 3d). Although cells and other phase objects are hardly visible in single pass bright-field microscopy, the outline of single cells becomes clearly visible in multi-pass microscopy images. Both absorption and phase shifts need to be taken into account in the analysis of such images and future high-resolution multi-pass microscopy studies will aim at exploring fine details in the interior of cells.

## Discussion

Multi-pass microscopy is a technique for signal amplification and allows for optimal parameter estimation in the presence of noise sources that are not significantly amplified by multi-passing (such as shot noise or read noise). For the images acquired at constant damage, the technique presented above relies on temporal post-selection of a few percent of the light that interacted with the sample. Although this degree of postselection is orders of magnitude less stringent than the postselection required in comparable N00N-state microscopy experiments, we note that there are at least two technologically feasible ways of overcoming this need for postselection: on the one hand, an optical switch could be incorporated into the cavity, which would allow for out-coupling all light at once. This could be achieved using a Pockels cell and a polarizing beam splitter. On the other hand, even without an out-coupling switch a metrological advantage can be gained, if a detector is used that records the detection time, and thus the number of interactions, of each individual photon.

Postselection free multi-pass microscopy will benefit applications that are sensitive to photo-induced damage, such as live-cell microscopy^16^, the label-free detection of single proteins^17^, the detection of single molecules via their absorption^18^ or low damage imaging of exotic quantum states of matter^19^. Apart from increasing the detectability of such weak signals^17^,^18^, an improved signal-to-noise ratio would also lead to better spatial accuracy in super-resolution techniques that rely on these weak signals. Increased accuracy is also obtained if a measurement is limited by the number of detectable particles, for example, at wavelengths where there are no high-intensity light sources, or also in measurements that involve massive particles as probe particles (electron or ion microscopy, measurements involving anti-matter). Multi-pass electron microscopy especially seems worth considering, as sample damage limits the spatial resolution achieved in imaging biological specimens^20^,^21^.

## Methods

### Experimental setup

Two different laser systems were used for the experiments:

For the data in Fig. 3, a titanium sapphire laser was used (Venteon, 10 fs pulse width, 5 nJ per pulse, 100 MHz repetition rate, spectrally centred at *λ*=780 nm). The pulses are spectrally filtered (Semrock LL01-810-12.5) to reduce the effect of chromatic aberrations in the self-imaging cavity. This inevitably broadens the temporal width of the pulses; however, the pulses are still short compared with the cavity round trip time. A resonantly driven electro-optic modulator is used to reduce the laser repetition rate to 50 MHz with an extinction ratio of about 16 dB.

A lower repetition rate laser was required for the data in Fig. 2, to allow for more interactions before the advent of the consecutive laser pulse. The laser diode (783 nm) was driven with fast voltage pulses. Pulse widths <1 ns at a peak power of about 100 mW were achieved. The repetition rate was controlled using a direct digital synthesizer (Novatech 409B) and set to 25 MHz.

The alignment of the self-imaging cavity is done element by element (starting from the out-coupling mirror) by maximizing the light that is back-reflected into the single mode fibre.

### Jones matrix representation of quartz-silica depolarizer

The quartz-silica depolarizer consists of a wedged plate of optical quartz cemented to a wedged plate of synthetic fused silica (OptoSigma DEQ 2S). The quartz crystal is cut and oriented such that it has the fast axis at a 45° angle with respect to the polarization of the incoming beam. The fused silica wedge has negligible birefringence and prevents beam deviation. The Jones matrix of the quartz crystal can be written as 
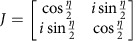
, where *η* is the phase retardance due to the birefringence of the crystal. The retardance is proportional to the thickness of the crystal, which varies spatially, as the crystal is cut with a wedge angle of *β*∼2°: *η*∼*β*Δ*x*Δ*n*/*λ*, where Δ*n*∼0.009 is the difference in index of refraction of light polarized along the fast or slow axis of the crystal. In the experiment horizontally polarized light 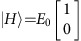 enters the multi-pass microscope (*I*_0_=|*E*_0_|^2^ is the intensity of the incoming light), interacts with the quartz crystal *m* times and is finally projected onto the vertical polarization axis and detected. This can be written as 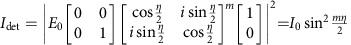.

### Measurement error in retardance measurements

The error in retardance measurements is obtained from error propagation as 
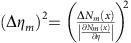
. For a constant number of detected particles the s.d. 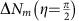 is independent of *m*, whereas the slope 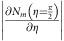 increases linearly with *m*. This leads to a 
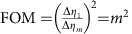
, which scales with the square of the number of roundtrips. If the number of probe particle–sample interactions is to be kept constant, for a lossless setup and sample, 
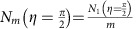
 and 
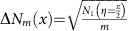
. The slope 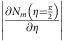 would still increase linearly with *m*, leading to a 
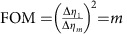
. For 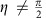, the setup could be adapted to reach optimal sensitivity^3^.

### Measurement error in absorption measurements

Assume *N*_0,*m*_ photons are used to probe the transmission of a sample. After *m* interactions, all photons are out-coupled from the cavity and detected with a photon counting detector. Without the sample, 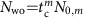 photons will be detected. Uncorrelated repetitions of the measurement of *N*_wo_ will give a s.d. of 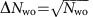. With a partially absorptive sample in the setup, 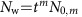 photons will be detected with a s.d. of 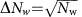. It follows that 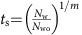 and error propagation yields a s.d. of the measured sample transmission of 
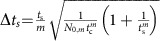
. We are interested in the s.d. as a function of *m* at constant damage. This implies that the number of in-coupled photons *N*_0,*m*_ will be a function of *m*, such that the number of absorbed photons D_*m*_ is independent of *m*. For a symmetric setup, in which the cavity losses are the same on both sides of the sample, we get 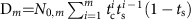 and D_*m*_=D_1_ yields 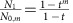 and


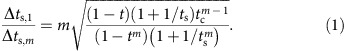


For *mα*<<1, this yields a FOM of multi-pass absorption microscopy 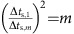 that scales linearly with *m*, just as it did for the retardance measurements.

When studying living samples, it might be more important to look at the damage reduction at constant s.d. (Δ*t*_s,1_=Δ*t*_s,*m*_, but D_*m*_≠D_1_), which yields


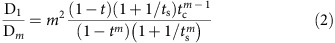


and scales with *m* for *mα*<<1.

### Image acquisition and noise analysis

To assess the single-pass shot-noise limit, the spatially resolved photon counting capabilities of the ICCD camera are exploited. it is noteworthy that they are not required to benefit from multipass microscopy.

For the retardance measurements in Fig. 2, the exposure is chosen such that ∼140 photons are detected per image. There are fewer than one dark counts per image. For each value of *m*, 10,000 images were acquired. This total data set, containing roughly 1.4 × 10^6^ detected photons, is divided into *N*_p_=100 images, each of them containing *N*_*m*_ photons, with *N*_1_=1.4 × 10^4^, which were randomly picked without replacement. For each of these *N*_p_ images, the 
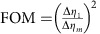
, where 
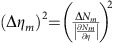
, is calculated and plotted in Fig. 2c. For this calculation, the slope 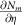 is obtained from a sinusoidal fit to the sum of all *N*_p_ images. The above equation can be rewritten as 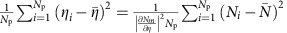 and, as the images are independent of each other, 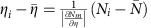. This allows us to calculate^22^ the s.d. of the variance as 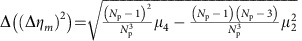, with *μ*_2_ and *μ*_4_ being the second and fourth central moment of the distribution of values 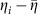. The s.d. of the 
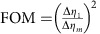
 is then obtained from propagating the errors in (Δ*η*_*m*_)^2^ and (Δ*η*_1_)^2^.

The equations for the calculation of *N*_*m*_ that are given in the main text assume a symmetric setup, with equal loss *α*_c_ on both sides of the sample plane. For the data and theory shown in Fig. 2, the asymmetry due to the unequal reflectance of *M*_i_ and *M*_o_ is taken into account when calculating *N*_*m*_ and *m*_*α*_. With *α*_i_=1−*t*_i_ and *α*_o_=1−*t*_o_ being the total losses on the in-coupling and out-coupling side of the sample, respectively, we get 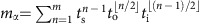 and 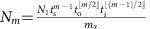 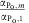, respectively. As the images in Fig. 2 are centred at 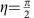, the ratio 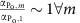, with a maximum measured deviation of 0.04. For the theoretical curves in Fig. 2c, 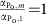 was assumed for even numbers of interactions, for which *α*_Po,*m*_ could not be determined experimentally.

For the optical density measurements in Fig. 3 the process is similar. At *m*=1, 100 images are recorded, each with a total integration time *τ*_1_. For *m*>1, the integration time is *τ*_*m*_=*τ*_1_/*m*_*α*_ to operate at constant damage. For each image, the total number of detected photons is integrated. The process is repeated without the sample and the transmission 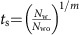 and its variance are calculated. The error bars in Fig. 3 corresponds to the s.d. of the FOM as obtained from error propagation. For the theory curve, the asymmetry of the setup is again taken into account, which can result in kinks in the FOM between odd and even interaction numbers.

### Data availability

The data that support the findings of this study are available from the corresponding author upon request.

## Additional information

**How to cite this article:** Juffmann, T. *et al*. Multi-pass microscopy. *Nat. Commun.* 7:12858 doi: 10.1038/ncomms12858 (2016).
